# The adolescent dysregulation profile and its association with educational, occupational, and substance use outcomes in emerging adulthood

**DOI:** 10.1177/01650254241308468

**Published:** 2024-12-25

**Authors:** Tara R. Cooper, Paweena Sukhawathanakul

**Affiliations:** University of Victoria, Canada

**Keywords:** Dysregulation profile, emerging adulthood, education, occupation, substance use

## Abstract

The dysregulation profile (DP) in youth is characterized by severe emotional, cognitive, and behavioral dysregulation and is associated with an increased risk for psychiatric disorders. Adolescent self-regulation has important implications for adulthood outcomes, yet this association is understudied in the context of the DP in emerging adults. Using a Canadian adolescent sample (*N* = 662; *M*_age_ = 15.52), a dimensional approach to the DP was taken and associations between adolescent DP scores and educational, occupational and substance use outcomes in emerging adulthood (*M*_age_ = 25.77) were examined. Latent profile analysis revealed: a highly problematic group with elevated DP scores (*n* = 59; 8.9%); an average problems group with average DP scores (*n* = 285; 43.1%); and a low problems group with below average DP scores (*n* = 318; 48%). The highly problematic group reported the lowest occupational statuses, and the highest prevalence of unemployment and substance dependencies. These results demonstrate the impact of subthreshold adolescent DP scores on emerging adulthood outcomes.

The dysregulation profile (DP) in children and adolescents is characterized by severe cognitive, emotional and behavioral dysregulation. Youth with the DP are at a higher risk for a range of adjustment problems including suicidality, poor psychosocial functioning, and greater likelihood of a psychiatric disorder diagnosis (Althoff et al., 2010; Biederman et al., 2012; Deutz et al., 2018). Scores above 210 points (i.e., two standard deviations above the average) on the anxious/depressed, attentional problems, and aggressive behaviors syndrome scales (AAA scale) of the Child Behavioural Checklist (CBCL; Achenbach, 2011) define this behavioral phenotype (Althoff et al., 2010). While the extant literature on the DP have predominantly focused on the DP as a diagnostic category and its relationship to psychopathology, little is known about the relationship between subthreshold DP scores and functional outcomes such as educational attainment and occupational success which are important developmental tasks of young adulthood. Youth with higher DP scores may have enduring problems in young adulthood including problems with self-monitoring, planning, decision-making, persisting with complex tasks (e.g., applying for work, completing required coursework), managing frustrations effectively when tasks are challenging, as well as maintaining supportive relationships to help cope with stressors. Emotional, behavioral, and cognitive regulation in these domains can ease the transition into post-secondary education and/or the workforce. This study examines how dysregulation below the threshold for the DP in adolescence relates to educational and occupational outcomes in emerging adulthood.

## The DP and Psychiatric Disorders in Childhood and Adolescence

The DP is conceptualized as a latent construct composed of severe deficits in three domains and shares meaningful relationships with various outcomes. For instance, the DP in childhood and adolescence predicts a range of psychiatric disorders in young adulthood. Biederman et al. (1995) first investigated the predictive validity of the DP and found it was strongly associated with juvenile bipolar disorder (J-BPD) among children. However, subsequent studies have associated the DP to a wider range of psychiatric outcomes (Aebi et al., 2020; Althoff et al., 2010; Deutz et al., 2018; Dolitzsch et al., 2016; Holtmann et al., 2011; Thompson et al., 2011; Wang et al., 2018). For instance, prior to diagnosis of a psychiatric disorder, Althoff et al. (2010) observed that participants between 3 and 17 years of age with the DP were at a significantly higher risk for any psychiatric disorder 14 years later when compared with the group with low DP scores. Likewise, Deutz et al. (2018) found that the DP measured in early childhood, childhood or adolescence predicted 12 out of 18 personality psychopathologies 3 years later. Finally, when the DP co-occurs with psychiatric disorders, children and adolescents have significantly poorer psychiatric outcomes than those with only a psychiatric disorder. For example, Biederman et al. (2012) found that children with attention-deficit/hyperactivity disorder (ADHD) *and* the DP had significantly higher rates of psychiatric hospitalizations and poorer psychosocial functioning 7 years later. Similar negative outcomes have been found when the DP co-occurs with bipolar disorder, major depressive disorder or conduct disorder (Biederman et al., 2009).

The co-morbidity between the DP and psychiatric disorders may be explained by their shared risk factors. In terms of biological risk factors, Ayer et al. (2013) found that adolescents with the DP had significantly more blunted hypothalamic-pituitary adrenal (HPA) axis responses to a stressful task compared with those with below average DP scores after controlling for socioeconomic status (SES), body mass index (BMI), pubertal stage and experiences of abuse. Since HPA dysfunction is implicated in psychiatric disorders such as depression, bipolar disorder, schizophrenia, and borderline personality disorder (Ayer et al., 2013; Burke et al., 2005; Nater et al., 2010; Steen et al., 2011) and the DP is a harbinger for various psychiatric disorders (Aebi et al., 2020; Althoff et al., 2010; Deutz et al., 2018; Dolitzsch et al., 2016; Holtmann et al., 2011; Thompson et al., 2011; Wang et al., 2018), the comorbidity between the DP and psychiatric disorders may be explained by their shared association with HPA dysfunction. Other studies have also identified genetic vulnerabilities that relate the DP with other psychiatric disorders. In a genomic linkage study, 71% of variability in DP scores was attributed to inherited genetic factors (Doyle et al., 2010) which were also associated with other psychiatric disorders including bipolar disorder, schizophrenia, autism and ADHD, suggesting that the DP is associated with other psychiatric disorders through similar genetic risk factors.

The DP also shares similar contextual risks factors with other psychiatric disorders. For example, in a sample of 6,100 children, Basten et al. (2013) found that developing in a context in which parents had serious psychological symptoms was related to significantly higher likelihood of the DP. Furthermore, family environments characterized by rejection, neglect or abuse place children and adolescents at higher risk for the DP (Aebi et al., 2020; Maguire et al., 2015; Thompson et al., 2011). In a sample of Swiss children and adolescents, 12% of youth in the foster care system met the criteria for the DP (Dölitzsch et al., 2016) whereas the estimated prevalence of the DP in community samples was 1% according to Holtmann et al. (2011). Likewise, in a sample of adolescents and emerging adults, Aebi et al. (2020) found that life adversities and parental rejection were significant risk factors for the DP 7 years later. Similar results have been observed in the literature on children’s risk for psychiatric disorders in the context of parental psychopathologies, negative parenting styles or harsh family environments (Ashman et al., 2002; Beidel & Turner, 1997; Biederman et al., 2001; Goodman & Gotlib, 1999). Therefore, the DP and various psychiatric disorders may share common environmental and biological risk factors.

## The DP and Behavioral Outcomes

Studies have found that the DP is associated with a range of maladaptive behavioral outcomes including substance abuse and suicidality. In the context of substance use and dependencies, the DP is associated with risks for substance use disorders after controlling for other psychiatric disorders in adolescents and young adults (Althoff et al., 2010; Holtmann et al., 2011; Thompson et al., 2011). For example, independent of a concurrent psychiatric disorder, Althoff et al. (2010) found that the risk of drug abuse 14 years later for children with the DP was 11 times higher than for children with low DP scores. Similarly, in a sample of children followed over 19 years, Holtmann et al. (2011) found that the DP significantly predicted problematic alcohol and cannabis use and dependence in young adulthood after controlling for psychiatric disorders.

DP scores measured in childhood also independently predict the risk of suicidal behavior at age 19, above and beyond the presence of psychiatric disorders (Holtmann et al., 2011). When the predictive ability of DP scores is compared with the predictive ability of its syndrome scales, DP scores predicted self-harm and suicidal ideation whereas each of its syndrome scales did not relate to such outcomes (Deutz et al., 2016). Therefore, the DP in childhood is related to a range of negative outcomes in adolescence and emerging adulthood.

## A Dimensional Approach to the DP

Although the categorical definition of the DP may be useful for clinical purposes, it fails to recognize that a continuum exists between normal scores and the DP. Previous research indicates that subthreshold dysregulation relates to meaningful psychopathological outcomes. For instance, children with DP scores between one and two standard deviations above the mean demonstrate significantly higher rates of anxiety and disruptive disorders than children with normal DP scores (Spencer et al., 2011). This profile of scores is conceptualized by others as deficient emotional self-regulation (DESR; Spencer et al., 2011; Surman et al., 2013). When intermediate DP scores co-occur with ADHD, children present with significantly more impairments in psychosocial functioning than ADHD children with normal DP scores (Spencer et al., 2011).

Critically, compared with children who meet the criteria for the DP, those whose scores are consistent with DESR have a less severe risk for a variety of outcomes related to adjustment (Biederman et al., 2012; Soler-Gutiérrez et al., 2023). For instance, Biederman et al. (2012) found that 50% of adolescents with the DESR reported severe psychosocial impairment as opposed to 64% of adolescents with the DP. Furthermore, 9% of adolescents with the DESR reported psychiatric hospitalization compared with 21% of adolescents with the DP (Biederman et al., 2012). Finally, Biederman et al. (2012) found that the risk for substance use disorder was significantly higher for adolescents with the DP compared with those with the DESR.

Given that subthreshold DP scores continue to relate to meaningful psychopathological outcomes, the categorical definition of the DP may draw an arbitrary line in the sand. In fact, the majority of psychopathologies fall below the threshold for classification and yet, still relate to meaningful symptom burden (Linscott & van Os, 2013). Therefore, a dimensional approach to dysregulation could overcome the limitations of a tradition diagnostic approach by examining the outcomes related to subthreshold levels of dysregulation and substantiating the dimensional approach to psychopathology, such as that proposed by the Hierarchical Taxonomy of Psychopathology (HiTOP; Kotov et al., 2022).

## Educational and Occupational Outcomes and the DP

Extant literature on the DP has often focused on the risks for psychiatric disorders and mental health outcomes but other functional outcomes in the domains of educational and occupational attainment have not received attention. As adolescents mature into young adults, demands from the environment increase in complexity and scope (Turgay et al., 2012). To meet these demands, adolescents must recruit internal and external resources. For adolescents with high DP scores, internal resources, such as their emotional regulation, may not be adequate for the demands of occupational and academic environments. Likewise, as external resources provided by parents or guardians wane, adolescents with high DP scores may not be able to achieve fundamental development tasks such as completing education and securing employment (Masten et al., 1999). Such resource deficits during the critical transition from adolescence to emerging adulthood may negatively affect the accumulation of crucial life experiences for individuals with high DP scores. Furthermore, given that relationship formation is an important task of emerging adulthood, fewer educational or occupational experiences may limit individuals’ opportunities to socialize with others and potentially make lasting friendships, which could in turn, impair an individual’s ability to cope with their educational or occupational challenges. Since high DP scores are more prevalent in individuals with attentional problems compared with individuals without attentional concerns (Biederman et al., 2012), exploring the functional outcomes associated with a diagnosis of childhood ADHD may elucidate the functional outcomes likely to co-occur with high DP scores.

A review of seven longitudinal studies on the lifespan outcomes for children diagnosed with ADHD consistently found that educational underachievement was characteristic across the samples (Cherkasova et al., 2022). Those with ADHD were more likely to repeat grades, complete less education, not attend or complete college, and require remedial educational services when compared with those without ADHD (Barkley & Fischer, 2019; Cherkasova et al., 2022; Loe & Feldman, 2007; Owens et al., 2017). Furthermore, in terms of occupational attainment, children whose ADHD persisted into adulthood were more often unemployed or had unstable employment histories (Able et al., 2007; Barkley & Fischer, 2019; Owens et al., 2017). Results from studies using the Hollingshead Social Status scale report that adults with ADHD have significantly lower SESs than their peers and their family of origin (Barkley & Fischer, 2019; Biederman et al., 1993). Due to the co-occurrence of high DP scores with ADHD, these findings suggest that individuals with high DP scores may also experience greater educational and occupational difficulties in emerging adulthood.

The purpose of this study is to examine DP scores in a Canadian sample of youth 12–19 years old. Unlike the general psychopathology factor previous reported in the literature, the DP was selected because of its unique specification of attention problems. Deutz et al. (2020) compared the predictive ability of the DP and the P-factor and found that the attention problems subfactor of the DP uniquely predicted academic achievement. These results emphasize the unique importance of attention in functional contexts such as education and may also reveal important differences in the context of occupation. Therefore, the DP was selected instead of the P-factor to address our research questions with respect to educational and occupational attainment.

Based on previous observations of high scores consistent with the DP and low DP scores in representative samples of adolescents (Aebi et al., 2020; Althoff et al., 2010; Wang et al., 2018), and high internalizing and externalizing problems characteristic of female and male adolescents, respectively (Bongers et al., 2003), we predicted there would be four groups in the sample. Youths’ adolescent DP scores (at age 12–19) were used to assess educational and occupational outcomes in emerging adulthood when youth were between 22 and 28 years.

A person-centered approach using latent profile analysis (LPA) was used to characterize the heterogeneity of DP scores. This analytic approach offers a unique opportunity to describe group patterns in a population whose distributions are expected to be heterogeneous. Unlike a mean difference approach which restricts data to a normal distribution, LPA allows an investigation of different population distributions within the sample (Nagin, 1999). As observed in previous studies, DP scores are typically distributed on a censored normal distribution; a sizable cluster of individuals score at the minimum end of each syndrome scale while a smaller contingent of individuals score at the maximum end of each syndrome scale (Aebi et al., 2020; Althoff et al., 2010; Wang et al., 2018). Rather than assuming that all individuals’ scores are normally distributed, LPA provides the opportunity to explore distributional heterogeneity within a population (Nagin, 1999). Previous researchers have used LPA to describe patterns of DP scores related to early risk factors in childhood or psychiatric outcomes in adulthood (Aebi et al., 2020; Althoff et al., 2010; Basten et al., 2013; Wang et al., 2018). This study extends the existing literature on DP scores using LPA to describe corresponding educational and occupational outcomes in emerging adulthood.

## Methods

### Participants

Participants were part of the Victoria Healthy Youth Survey (V-HYS), a comprehensive survey of Canadian youth in the city of Victoria, British Columbia, Canada. Using a random sample of 9,500 private telephone listings, 1,036 adolescents and their parents or guardians were eligible to participate in 2003 (Leadbeater et al., 2012). A total of 662 adolescents (342 females) consented to participate in the study at baseline. Adolescents at baseline were between 12 and 19 years old. The demographics of the sample at baseline consisted of 85% Caucasians, 4% Asians, 4% multiracial, and 3% Aboriginals (see Table 1). The remaining 4% belonged to other ethnic groups (e.g., Hispanic, Black, or other). Ethnicity, parental levels of education and living arrangements of the sample were representative of the population from which the sample was drawn (Albrecht et al., 2007). Due to attrition from baseline in 2003 to follow-up in 2013, data on occupational, educational and substance use related emerging adulthood outcomes was only available for 474 participants (71.6%).

**Table 1.** Demographic Characteristics and Bivariate Correlations Between Syndrome Scales.

Characteristic	Value	Range	Anxious/depressed syndrome	Attention problems syndrome
Sex at baseline, *n* (%)				
Female	342 (51.7%)			
Male	320 (48.3%)			
Age at baseline, mean (*SD*)	15.52 (1.93)	12–19		
Age at follow-up, mean (*SD*)	25.77 (1.96)	22–28		
White/Caucasian, *n* (%)	561 (84.7%)			
Asian/Asian-Canadian, *n* (%)	28 (4.2%)			
Mixed/Bi-Racial, *n* (%)	25 (3.8%)			
Indigenous, *n* (%)	20 (3.0%)			
Other, *n* (%)	27 (4.3%)			
Anxious/depressed syndrome, mean (*SD*)	8.60 (4.22)	0–23		
Female, mean (*SD*)	9.06 (4.46)	0–23		
Male, mean (*SD*)	8.10 (3.89)	0–23		
Attention problems syndrome, mean (*SD*), *r*	4.67 (2.43)	0–12	.42*** [.35, .48]	
Female, mean (*SD*)	4.51 (2.53)	0–12		
Male, mean (*SD*)	4.84 (2.31)	0–12		
Aggressive problems syndrome, mean (*SD*), *r*	5.29 (3.23)	0–23	.45*** [.38, .51]	.49*** [.43, .55]
Female, mean (*SD*)	5.17 (3.27)	0–23		
Male, mean (*SD*)	5.42 (3.18)	0–23		

*Note. M* and *SD* are used to represent mean and standard deviation, respectively. Values in square brackets indicate the 95% confidence interval for each correlation. The total sample size was 662.

* *p* < .05; **p < .01; ***p < .001.

### Procedure

The V-HYS was administered to participants at baseline in 2003 and at follow-up in 2013 by a trained interviewer at the subject’s homes or another location that afforded privacy (e.g., the University of Victoria). Informed consent was obtained from participants and parents or guardians at baseline and only by the participants at follow-up. The survey consisted of a 2-part questionnaire that addressed sociodemographics; family, peers and school environment; mental health; and substance use. Part 1 was read to the participants and answers were recorded by the interviewer. Part 2 was self-report, completed by the participants to provide the participants with confidentiality in their responses to sensitive issues such as illegal substance use. Participants were compensated with a $35 CND honorarium at baseline and again at the follow-up. The study was approved by the Research Board of Ethics at the University of Victoria (protocol number 09-292).

### Educational Attainment

Educational attainment was self-reported by participants at follow-up. 477 participants had data for this variable. Participants were asked to report the highest level of education completed at the time of data collection using a 5-point scale: High School or less; some training college or trade; trade certificate or diploma; college/university certificate or diploma; or Bachelor or higher. For the purpose of analysis, the educational attainment scale was condensed into a 3-point scale: High school or less; some training college or trade, trade certificate or diploma; and college/university certificate, diploma, bachelor or higher.

### Occupational Attainment

Occupational attainment was self-reported by participants using the Hollingshead Occupational Status Scale at follow-up (Bornstein et al., 2003; Hollingshead, 1975). 466 participants had data for this variable. Participants were asked to report the highest level of occupational attainment at the time of data collection by selecting one of the nine categories: (1) menial service workers (e.g., cleaner, produce worker); (2) unskilled worker (e.g., cashier, server); (3) semi-skilled workers (e.g., child care worker, roofer); (4) skilled workers/small business (e.g., administrative assistant, receptionist); (5) clerical and sales workers (e.g., book keeper, medical office assistant); (6) technicians/semi-professionals (e.g., lab technician, human resource officer); (7) managers/lesser professionals (e.g., teacher, social worker); (8) administrators/minor professionals (e.g., registered nurse, registered massage therapist); and (9) executives/major professionals (e.g., doctor, veterinarian). Unemployed participants were also coded in this variable. For the purpose of analysis, the scale was condensed into four categories: (1) menial, unskilled and semiskilled workers; (2) skilled workers/small business, clerical/sales workers and technicians/semi-professionals; (3) managers, lesser/minor professionals, administrators, executives, major professionals; and (4) unemployed.

### Substance Use

Alcohol and cannabis abuse or dependence was assessed using the Mini-International Neuropsychiatric Interview (MINI) (Sheehan et al., 1998). Participants were asked to reflect on their alcohol or cannabis use over the past 12 months and answer a series of questions regarding their substance use and its impact on daily living. Alcohol dependence was defined when participants reported they used alcohol in the past year and answered yes to three or more of the questions in the MINI section on alcohol dependence. 470 participants had data for alcohol dependence. The same criteria were used to determine cannabis dependence and 474 participants had data for this variable. Participants who had a positive diagnosis for alcohol or cannabis dependence were assigned the value 1 whereas participants who had a negative diagnosis were assigned the value 0. Coders ensured that dependency was ruled out first, prior to assessing abuse. Abuse was defined when participants reported they used alcohol in the past year and answered yes to 1 or more of the questions in the MINI section on alcohol abuse. 661 participants had data for alcohol abuse. The same criteria were used to assess cannabis abuse and 474 participants had data for this variable. Participants who had a positive diagnosis for alcohol or cannabis abuse were assigned the value 1 whereas participants who had a negative diagnosis were assigned the value 0.

### Behavioral Problems

Behavioral problems were measured using the Child Behaviour Checklist (CBCL/6-18; Achenbach, 2011) which consists of 118 problem behaviors likely to occur in children aged 6 to 18 years. The 118 questions can be reduced to eight narrow band syndrome scales including the anxious/depressed, aggressive behaviors and attention problems syndrome scales. Consistent with previous studies (Aebi et al., 2020; Althoff et al., 2010; Basten et al., 2013; Biederman et al., 2012; Holtmann et al., 2011; Rescorla et al., 2021), scores on the following syndrome scales were used to define the DP: attention problems (α = .71), anxious/depressed (α = .86) and aggressive behaviors (α = .78). Raw scores on each syndrome scale were converted into a standardized T-score, where a score of 50 indicated average functioning and every 10 points above or below the mean represented one standard deviation. Each syndrome T-score was used to conduct the LPA. Separate from the LPA, the T-scores on each syndrome scale (i.e., attention problems, anxious/depressed, and aggressive behaviors) were summed to compare the total DP score with the total DP scores previously reported in the literature (Aebi et al., 2020; Biederman et al., 2012; Holtmann et al., 2011).

### Analytic Strategy

LPA was used to identify high DP scores based on T-scores on three syndrome scales of the CBCL assessed during adolescence when youth were 12 to 19 years of age. The optimal number of profiles estimated was based on model comparisons approach using the following decision criteria to derive the best fitting model: relatively low Bayesian information criterion (BIC) and Akaike information criterion (AIC) values, and entropy values equal to .80 or greater (Ferguson et al., 2020). Differences in substance use, educational and occupational attainment in young adulthood when youth were 22 to 28 years of age were then assessed based on the different types of behavioral profiles. Chi-square analyses or a Fisher’s exact tests in R were conducted to assess for group differences. The preregistration for this study can be viewed on Open Science Framework: https://osf.io/cuwpk/.

## Results

Demographic characteristics of the sample are presented in Table 1. Female participants (342; 51.7%) had significantly higher scores on the anxious/depressed syndrome scale (*Mdn* = 9, *SD* = 4.46) than males (*Mdn* = 8, *SD* = 3.89), *W* = 47745, *p* = .005. Males had significantly higher scores on the attention problems syndrome scale (*Mdn* = 5, *SD* = 2.31) than females (*Mdn* = 4, *SD* = 2.53), *W* = 59378, *p* = .05. No significant sex differences were observed on the aggressive behavior syndrome scale.

Correlations between the anxious/depressed, attentional problems and aggressive behaviors syndrome scales are presented in Table 1. Attentional problems shared a significant and strong positive correlation with the anxious/depressed (*r* = .42, *p* < .001) and with the aggressive behaviors syndrome scales (*r* = .45, *p* < .001). Likewise, the aggressive behaviors scale shared a significant and strong, positive correlation with the anxious/depressed syndrome scale (*r* = .49, *p* < .001). Due to attrition from baseline in 2003 to follow-up in 2013, data on occupational, educational and substance use related emerging adulthood outcomes was only available for 474 participants. Participants who remained in the study had significantly lower scores on the attention problems syndrome scale at baseline (*M* = 4.49, *SD* = 2.39) than participants who dropped out (*M* = 4.97, *SD* = 2.47), *W* = 56,163, *p* = .01.

### DPs

**Figure 1.**
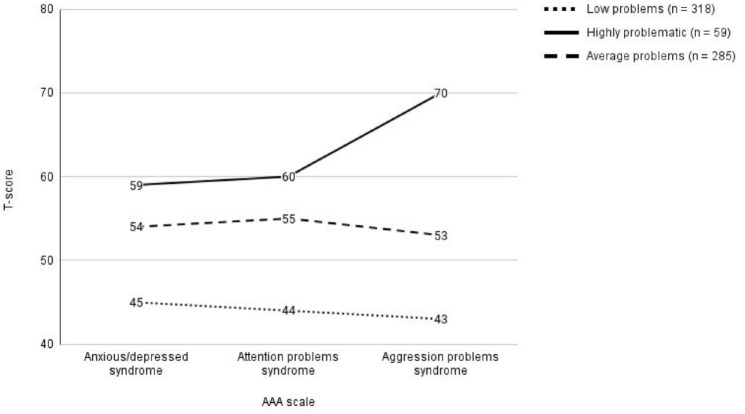
Mean T-Scores from the Latent Class Analysis with three Classes. AP = Attention problem syndrome scale, AD = anxious/depressed syndrome scale, AGG = Aggression problems syndrome scale. Derived classes from the LPA analysis. 50 is the mean score on each syndrome scale of the entire sample and the standard deviation of the entire sample is 10.

The model fit indices indicated that a three-class profile fit the data best. BIC and AIC values decreased from the one-class profile to the three-class profile and then increased in the four-class profile (see Table 2). Similarly, entropy indicated that the three-class profile was best at separating the classes. Figure 1 and Table 3 show the mean T-scores and sex distribution for each class in the three-class profile. There were no significant sex differences across the three classes.

**Table 2.** Model Fit Indices.

Classes	AIC	BIC	Entropy
1	14,768.56	14,795.52	1.00
2	14,464.13	14,509.07	.67
3	14,387.38	14,450.29	.70
4	14,401.55	14,482.44	.63

*Note.* The best model fit according to the AIC and BIC has three classes.

**Table 3.** Class Characteristics.

T-scores	Low problem (*n* = 318, 54.9% F)	Highly problematic (*n* = 59, 52.5% F)	Average problem (*n* = 285, 48% F)	Range
Total, mean (*SD*)	131.4 (7.45)	189.6 (7.45)	161.2 (7.45)	98.14–265.10
Aggressive problems, mean (*SD*)	43.2 (6.0)	70.2 (6.0)	52.8 (6.0)	33.61–104.92
Anxious/depressed, mean (*SD*)	44.5 (8.4)	59.3 (8.4)	53.8 (8.4)	29.62–84.12
Attention problems, mean (*SD*)	43.7 (7.9)	60.1 (7.9)	54.6 (7.9)	30.80–80.17

*Note.* Lower scores indicate fewer behavioral problems. The standard deviations (SDs) for each class are equal due to the assumption of equal variance in the latent class analysis. There were no significant sex differences between the classes. The total sample size was 662.

The first class represented the majority (48%) of the sample and displayed below average T-scores on all three syndrome scales and was subsequently labeled the “low problem class.” The second class represented an “average problem” class characterized by average scores on each of the syndrome scales. This class consisted of 43.1% of the sample. Finally, a class characterized by above average scores across the three syndrome scales consisted of 8.9% of the sample. Participants in this class had an average summated T-score of 189.6 points and scored especially high on the aggressive behaviors syndrome scale (*M* = 70.2, *SD* = 6.0, see Figure 1 and Table 2). This group was labeled the “highly problematic” class.

### Group Associations with Substance Use, Educational and Occupational Attainment

**Figure 2.**
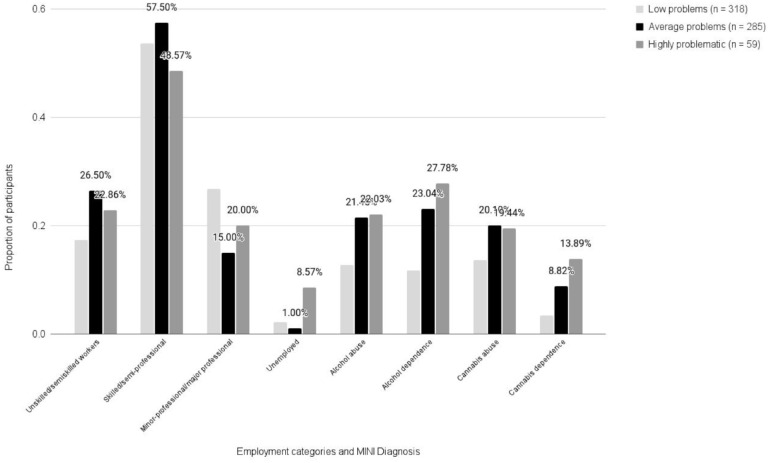
Distribution of Occupational Attainment and MINI Diagnosis Across the Latent Classes. Compared with participants in the other classes, a large proportion of participants in the highly problematic class were unemployed. The highly problematic class is highly represented in each of the four Mini-International Neuropsychiatric Interview (MINI) Diagnosis categories. The low problem class has significantly lower proportions of participants in each of the four MINI diagnosis categories.

Differences in emerging adult (i.e., when youth were 22–28 years old) substance use, educational, and occupational outcomes between the DPs were examined using Chi-square and Fisher’s exact test. There were notable differences across the classes. After correcting for multiple comparisons, participants in the low problem class were significantly less likely to have a positive MINI diagnosis for alcohol abuse compared with the other classes, χ^2^(2, 661) = 8.84, *p* = .01. Specifically, 12.8% of participants had a positive diagnosis for alcohol abuse in the low problem class compared with 22% of participants in the highly problematic class and 21.5% of participants in the average problem class (see Figure 2). The low problem class was also significantly less likely to have a positive diagnosis for alcohol dependence, χ^2^(2, 470) = 12.01, *p* = .002. Specifically, 12% of participants in the low problem class were alcohol dependent whereas 26.5% of individuals in the highly problematic class and 23.6% of individuals in the average problem class were alcohol dependent (see Figure 2). There were no significant differences between groups with respect to cannabis abuse at follow-up; however, according to Fisher’s exact test, the highly problematic class was significantly more likely to be dependent on cannabis (*p* = .008); 13.9% of the highly problematic class were cannabis dependent compared with 3.4% of the low problem class and 8.8% of the average problem class (see Figure 2).

In terms of occupational outcomes, Fisher’s exact test detected significant differences between classes at follow-up (*p* < .001). Among the participants who were employed, the majority of participants in each class attained the second category of occupation: skilled workers/semi-professionals. Specifically, 53.7% of the low problem class, 57.5% of the average problem, and 48.6% of the highly problematic class were skilled workers, sales workers or semi-professionals at follow-up (see Figure 2). Other differences between classes were observed at the highest level of occupational attainment: 26.8% of the low problem class attained the level of minor/major professionals whereas 15% of the average problem class had attained comparable levels of occupation (see Figure 2). Among the highly problematic class, 20% of participants attained the highest level of occupation whereas 22.9% attained the lowest level of occupation (see Figure 2). Therefore, the low problem class was most likely to attain the highest level of occupation whereas the average problem class was most likely to attain the lowest and the majority of all three classes attained the second level of occupation at follow-up.

Although the highly problematic group was expected to have the highest prevalence of low occupational attainment, a logistic regression revealed that the odds of missing occupational data, as opposed to non-missing occupational data, increased by 7% for each 1-point increase of attention problems in adolescence (*p* = .08). Therefore, missing data related to higher attention problems, characteristic of the highly problematic class, may have obscured the expected association between the highly problematic class and low occupational attainment.

Among the participants who were unemployed at follow-up, significant group differences emerged for the highly problematic class for whom 8.6% of participants were unemployed. In contrast, only 2.2% of the low problem class and 1% of the average problem class were unemployed at follow-up (see Figure 2). Therefore, the highly problematic class was significantly more likely to be unemployed at follow-up than the low problem or average problem class. With regard to educational outcomes, significant differences were not observed between the three classes across the three levels of educational attainment.

## Discussion

Previous literature have consistently reported a strong association between the DP and various psychiatric disorders, suicidal behavior and substance use (Althoff et al., 2010; Basten et al., 2013; Bellani et al., 2012; Biederman et al., 2012; Holtmann et al., 2011). However, the impact of subthreshold DP scores on educational or occupational outcomes in emerging adulthood has been relatively understudied. This study describes how subthreshold DP scores relate to these functional outcomes in emerging adulthood in a sample of Canadian adolescents.

Findings from the latent class analysis revealed heterogeneity in DP scores characterized by varying levels of anxious/depressed, attentional problems and aggressive behaviors. Three groups were identified: a highly problematic group characterized by high scores on the anxious/depressed and attentional problems scales and especially high scores on the aggressive behaviors scale (8.9%); an average problems group characterized by average DP scores (43.1%); and a low problems group characterized by below average DP scores (48%). It was predicted that a four-class model would fit the data based on previous observations of high and low DP in representative samples of adolescents (Aebi et al., 2020; Althoff et al., 2010; Wang et al., 2018), and high internalizing and externalizing problems characteristic of female and male adolescents, respectively (Bongers et al., 2003). However, a three-class model fit the data best which could be explained by the sample size of this study. In the study by Wang et al. (2018), two latent classes were observed, where sample size equaled 554. In studies with larger sample sizes, such as that of Aebi et al. (2020), six latent classes were found for a sample size of 1964 children. Similarly in the study by Althoff et al. (2010), a seven latent class model was found for a sample size of 2076 children. Therefore, the relatively smaller sample size in this study could explain the three-class model fit. Despite this, the pattern of the observed DP in the highly problematic group was consistent with previous results such that aggressive behaviors were the highest relative to the other two syndrome scales (Althoff et al., 2010; Basten et al., 2013; Rescorla et al., 2021).

The highly problematic group was characterized by scores above 60 points on the anxious/depressed and attentional problems syndrome scales and especially high scores on the aggressive behaviors syndrome scale (see Figure 1). The average summated T-score of the highly problematic group is reminiscent of the DESR profile (Biederman et al., 2012; Surman et al., 2013) which likely reflect the community sample distribution. DESR is a meaningful yet less severe risk factor for a variety of outcomes compared with the DP (Biederman et al., 2012; Soler-Gutiérrez et al., 2023). Consistent with these past studies, the highly problematic group observed in this study resembled the DESR as they were significantly more likely to have a diagnosis for alcohol abuse, and alcohol and cannabis dependence in emerging adulthood compare with the other latent groups. The especially high scores on the aggressive behaviors syndrome scale supports previous findings that externalizing problems (encompassing aggressive behaviors) in childhood or adolescence predicts substance use disorders later in life (Meque et al., 2019; Robson et al., 2020).

In addition to associations with substance use and dependency, those in the highly problematic group were significantly more likely to be unemployed in emerging adulthood. This finding is consistent with the literature on occupational outcomes in adulthood among youth with a childhood diagnosis of ADHD (Able et al., 2007; Barkley & Fischer, 2019; Owens et al., 2017). Individuals with high DP scores in this study could have struggled to acquire a highly skilled occupation due to problems with self-regulation that includes important skills in planning, self-monitoring, persisting on challenging tasks, and managing emotions which are required to organize behavior and meet the demands of a job environment (Robson et al., 2020). Furthermore, as adolescents enter emerging adulthood, parental or guardian supervision wanes. Attenuation of these external resources could have impacted the occupational outcomes for those with high DP scores due to the lack of intervention and support afforded by parents and guardians.

Among those in the highly problematic group who were employed in young adulthood, the majority were most likely to be employed in the two lowest categories: skilled workers or unskilled/menial service workers. Although significant differences were not observed between the profiles and educational outcomes, it could be that those with high DP scores were not able to apply their educational attainment to secure higher levels of occupation due to the quality of their academic performance. Since the highly problematic group resembled the DESR profile described by Biederman et al. (2012), they might not have had severe enough behavioral problems to limit their educational attainment. Given that emotional regulation has been associated with academic success in young children (Graziano et al., 2007; Robson et al., 2020), it could be that the impaired educational attainment of the highly problematic group was not observed due to a narrowed measure for academic success.

In sum, a relationship between subthreshold DP scores and substance use or employment was supported by our findings. Although DP scores did not relate to striking differences in employment type or educational attainment, scores did distinguish between binary categories (i.e., employed/unemployed, substance abuse/dependence). Since those with higher dysregulation scores may be more argumentative or oppositional, measures that are sensitive to the quality of functioning in work environments or academic performance (e.g., length of time it takes to complete the degree, detentions, academic engagement factors, etc.) may enhance the ability of DP profiles to reveal important occupational and educational group differences.

The average problems group was characterized by average DP scores. While youth with this DP displayed fewer problems relative to the highly problematic DP group, findings revealed that this profile also had a high occurrence of substance use problems. Relative to the highly problematic group, the average problems group had the second highest prevalence of substance abuse and dependency which again, is consistent with the general literature linking dysregulated behaviors to substance use (Robson et al., 2020), and implying an association to substance use in emerging adulthood that increases in strength as behavioral problems increase in severity.

In terms of occupational attainment, the average problems group had the highest prevalence in the two lowest occupational categories relative to the other profiles and the lowest prevalence of unemployment. As previously mentioned, missing occupational data may have obfuscated the prevalence of lower occupational attainment in the highly problematic group. Therefore, the unexpected high prevalence of low occupational attainment in the average problems group could be explained by the fact that nonmissing data were associated with lower attentional problem scores, which was characteristic of the average problems group as opposed to the highly problematic group.

Finally, relative to the other profiles, the low problems group was characterized by low DP scores. This group was the least likely to abuse alcohol or be dependent on alcohol or cannabis. Furthermore, this group was the most likely to attain the highest level of occupation in emerging adulthood. The low problems group scored below average on each syndrome scale indicating that they demonstrated very few dysregulated behaviors. Consistent with previous research, fewer dysregulated behaviors could relate to better occupational success because efficient self-regulation enhances an individual’s ability to meet the demands of a job (Robson et al., 2020).

### Limitations and Implications for Future Research

A considerable strength of this study is that it spans from early adolescence to emerging adulthood. Despite this strength, there are some notable limitations to discuss. First, given the community nature of this sample, few participants (*n* = 7) had scores consistent with the DP (i.e., scores above 210; Biederman et al., 2009). Since Holtmann et al. (2011) observed that the expected prevalence of high DP in community samples should be 1%, the presence of seven participants with high DP in this study is in line with the expected prevalence in community samples. As with all data-driven methods, the hypothesis of four observed groups, including a group consistent with the DP, was limited by the current sample size. Furthermore, it is likely that the observed differences in occupational and educational attainment would be enhanced if this research were conducted in a clinical sample. Despite this limitation, subthreshold DP scores observed in this community sample still revealed important difference between employment status (i.e., employed/unemployed) and substance use. Future research may want to collect data from a sample enriched with a clinical population to observe group consistent with the DP and describe group differences in functional outcomes.

The measures used in this study were also a limitation as they were all self-reported and thus subject to respondent bias. A more comprehensive characterization of behavioral dysregulation could be corroborated by use of informant report. Finally, high DPs were parameterized based on scores on three out of the eight syndrome scales of the CBCL reported during adolescence when youth were 12 to 19. Although previous studies have reported considerable stability in the DP over this developmental period (e.g., Althoff et al., 2010; Deutz et al., 2018), it is possible that youth may move in and out for their respective profiles depending on different contextual stressors they encounter during this developmental period.

## Conclusion

This study demonstrated that higher DP scores in adolescence were related to more substance use problems and lower occupational attainment in emerging adulthood. These results bring attention to the importance of high DP in affecting functional outcomes beyond psychiatric disorders. Critically, the results substantiate a dimensional approach to psychopathology as subthreshold scores were still meaningfully related to functional outcomes for participants. Future research should explore the impact of DP scores on other domains of life such as friendships, romantic relationships and parenting since these social engagements gain importance as adolescents transition into emerging adulthood. It is possible that higher DP scores also relates to poorer social relationships, impairing the ability of those with higher DP scores to cope with their occupational challenges and substance use problems through limited social support.

The relationship between subthreshold dysregulated behavior and various functional outcomes could make DP scores in adolescence an important indicator for initiating primary preventions. Recent reviews suggest that family or school-based interventions are effective at reducing the frequency of substance use among adolescents (Das et al., 2016). Since adolescents with higher DP scores are at greater risk for substance use, interventions such as these may help mitigate such negative outcomes. Furthermore, occupational intervention programs initiated in adolescence can help prepare adolescents with behavioral problems for the workplace. According to previous employment program studies, adolescents with behavioral problems who participated in employment readiness programs were more likely to be employed after the intervention than those who did not participate (Bullis & Cheney, 1999; Gates et al., 2018). Therefore, interventions designed to reduce substance use and improve occupational outcomes could help adolescents with behavioral problems succeed as they transition into emerging adulthood.
